# Synthesis, Characterization and Antibacterial Studies of *N*-(Benzothiazol-2-yl)-4-chlorobenzenesulphonamide and Its Neodymium(III) and Thallium(III) Complexes

**DOI:** 10.3390/molecules22020153

**Published:** 2017-02-22

**Authors:** Lawrence Nnamdi Obasi, Uchechukwu Susan Oruma, Ibrahim Abdulrazak Al-Swaidan, Ponnadurai Ramasami, Chigozie Julius Ezeorah, Alfred Ezinna Ochonogor

**Affiliations:** 1 Department of Pure and Industrial Chemistry, University of Nigeria, Nsukka 410001, Nigeria; nnamdi.obasi@unn.edu.ng (L.N.O.); chigozie.ezeorah@unn.edu.ng (C.J.E.); alfred.ochonogor@unn.edu.ng (A.E.O.); 2 Department of Pharmaceutical Chemistry, College of Pharmacy, King Saud University, P.O. Box 2457, Riyadh 11451, Saudi Arabia; ialsuwidan@ksu.edu.sa; 3 Computational Chemistry Group, Department of Chemistry, Faculty of Science, University of Mauritius, Réduit 80837, Mauritius; ramchemi@intnet.mu; 4 Department of Applied Chemistry, University of Johannesburg, Doornfontein Campus, Johannesburg 2028, South Africa

**Keywords:** *N*-(benzothiazol-2-yl)-4-chlorobenzenesulphonamide, neodymium(III) and thallium(III) complexes, antibacterial activity

## Abstract

*N*-(Benzothiazol-2-yl)-4-chlorobenzenesulphonamide (NBTCS) was synthesized by condensation reaction of 4-chlorobenzenesulphonyl chloride and 2-aminobenzothiazole in acetone under reflux. Neodymium(III) and thallium(III) complexes of the ligand were also synthesized. Both ligand and metal complexes were characterized using UV-Vis, IR, ^1^H- and ^13^C-NMR spectroscopies, elemental analysis and molar conductance measurement. IR studies revealed that the ligand is tridentate and coordinates to the metal ions through nitrogen and oxygen atoms of the sulphonamide group and nitrogen atom attached to benzothiazole ring. The neodymium(III) complex displays a coordination number of eight while thallium(III) complex displays a coordination number of six. The ligand and its complexes were screened in vitro for their antibacterial activities against *Escherichia coli* strains (*E. coli 6* and *E. coli 13*), *Proteus* species, *Staphylococcus aureus* and *Pseudomonas aeruginosa* using the agar well diffusion technique. The synthesized compounds were found to be more active against the microorganisms screened relative to ciprofloxacin, gentamicin and co-trimoxazole.

## 1. Introduction

**Figure 1:**
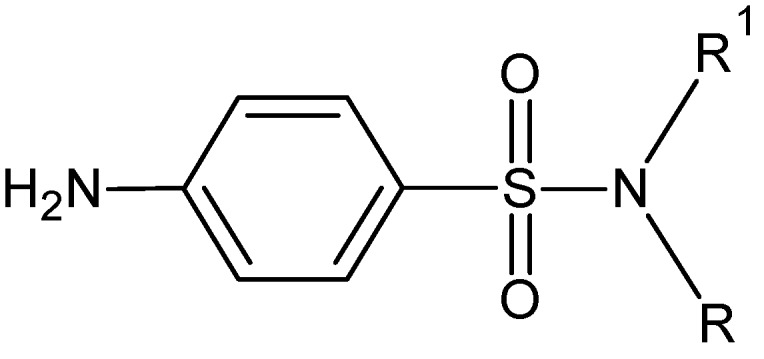
General structure of sulphonamides; R=R1=H for sulphanilamide.

Sulpha drugs, also known as sulphonamides, are compounds, which have a general structure represented by Figure 1. Sulpha drugs were discovered in 1930s during a series of studies with a bright red dye named prontosil [1,2]. Prontosil was found to be able to cure severe staphylococcal septicemia in human [2]. After the discovery of prontosil, several drugs containing the sulphonamide moiety have been prepared and put into diverse medical applications. They serve as antimicrobial drugs (sulphacetamide) [3,4,5]; diuretic drugs (acetazolamide) [6]; anticonvulsants (sultiame) [6]; dermatological drugs (mafenide) [7]; anticancer [8,9,10]; anti-inflammatory [11,12]; antihypertensive agent (bosentan) [13] and antiviral agents; and HIV protease inhibitors [14,15]. Their antimicrobial activities arise due to competitive inhibition of the enzyme dihydropteroatesynthetase (DHPS), an enzyme involved in folate synthesis. As such, the microorganism will be starved of folate and die. The similarity of the structure between sulphonamide and *p*-aminobenzoic acid (PABA), a molecule found in bacteria used in folic acid synthesis, is the basis for the inhibitory activity of sulpha drugs on dihydrofolate biosynthesis [1,6,16]. Sulpha drugs are associated with antibiotic resistance and allergies [17,18]. The work done by Yun et al. 2012 gave insight into sulpha drug resistance [1]. They revealed that the binding sites of PABA and sulpha drugs overlap, but those of sulpha drugs extend beyond the pocket in which PABA binds. Mutations associated with drug resistance cluster around this extended region of the PABA pocket. This explains how they can prevent the drugs from binding without seriously affecting the binding of PABA. Several modifications have been made on the general structure of sulphonamides, resulting in new compounds with varying pharmacological activities and reduced side effect [2]. The work done by Lawrence et al. 2010 have shown that the best therapeutic results were obtained from the compounds in which one hydrogen atom of the SO_2_NH_2_ group was replaced by heterocyclic ring [19]. Previous studies by Obasi et al. have led to discovery of ligands and complexes with varying activities against multi-resistant bacterial strains isolated under clinical conditions and cultured species using agar-well diffusion method [20,21,22]. Metal complexes of sulpha drugs have been reported with appreciable biological activities [23,24,25,26,27,28]. Recently, there have been several reports on neodymium(III) and thallium(III) complexes [29,30,31,32,33,34,35,36,37]. Mimouni et al., 2014 [38] have synthesized, characterized, and studied the antibacterial, antifungal and antitubercular activities of thallium complexes of monensin and lasalocid. Their study revealed that remarkable bioactivity against different pathogens were achieved and confirmed that the compounds could very likely be used without great risk of toxicity in diverse pharmaceutical applications. In addition, the crystal structures of thallium salt of Grisorixin and antibiotic 6016 thallium salt have been reported [39,40]. In view of the need for a new generation of antibiotics that would cause fewer side effects, we designed a new class of inhibitors namely: *N*-(benzothiazol-2-yl)-4-chlorobenzenesulphonamide (NBTCS) and its neodymium(III), and thallium(III) complexes. Their in vitro antibacterial activities against *Escherichia coli* strains (*E. coli 6* and *E. coli 13*), *Proteus* species, *Staphylococcus aureus* and *Pseudomonas aeruginosa* were also investigated.

## 2. Results and Discussion

**Scheme 1:**
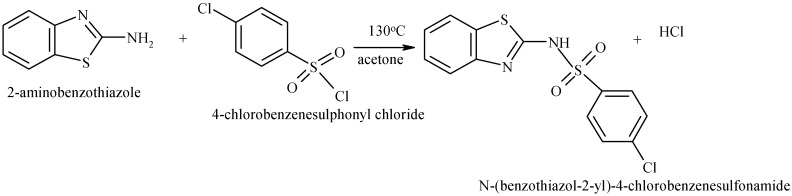
Synthesis of *N*-(benzothiazol-2-yl)-4-chlorobenzenesulphonamide.

The reaction of 2-aminobenzothiazole with 4-chlorobenzenesulphonylchloride yielded *N*-(benzothiazol-2-yl)-4-chlorobenzenesulphonamide (NBTCS) as shown in Scheme 1. The reaction of Nd(NO_3_)_3_·5H_2_O and C_6_H_9_O_6_Tl with NBTCS yielded [Nd(NBTCS)_2_(H_2_O)_2_]NO_3_ and [Tl(NBTCS)_2_]CH_3_COO, respectively. The ligand and complexes are air stable and have high melting points. They are soluble in ethanol, methanol, acetic acid and DMSO. The analytical data of NBTCS and its complexes are shown in Table 1. The elemental analysis of the NBTCS and its complexes show that the amount of carbon, hydrogen, nitrogen and sulphur (CHNS) are close to the experimentally determined values. The values of molar conductance of the complexes in DMSO at room temperature were found to be 72 Ω^−1^·cm^2^·mol^−1^ and 88 Ω^−1^·cm^2^·mol^−1^ for the Nd(III) and Tl(III) complexes, respectively. These values indicate 1:1 electrolytic nature of the complexes [41]. The ligand was found to be non-electrolyte with a molar conductance value of 15.6 Ω^−1^·cm^2^·mol^−1^.

### 2.1. Electronic Spectra

Electronic absorption spectra of the ligand and complexes in ethanol were recorded in the range 200–700 nm and the results are presented in Table 2. The electronic absorption spectra of the compounds show absorption bands between 230 and 282 nm, assigned to π–π* transitions. The band at 305 nm is attributed to n–π* transition from conjugation between the lone pair of electrons on p-orbital of nitrogen atom and conjugated π-bond of the benzene ring [42]. In the complexes, blue shifts were observed in the π–π* transition which could be as a result of removal of lone pair of electron on the electronegative atoms of the ligand upon coordination [43]. The Nd(III) complex did not contribute appreciably to the electronic spectra since for lanthanide ions f–f transitions are Larporte-forbidden and weak in nature.

### 2.2. Infrared Spectra

**Figure 2:**
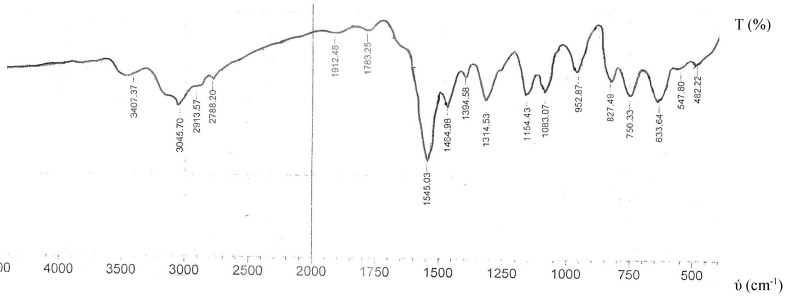
IR spectrum of *N*-(Benzothiazol-2-yl)-4-chlorobenzenesulphonamide (NBTCS).

**Figure 3:**
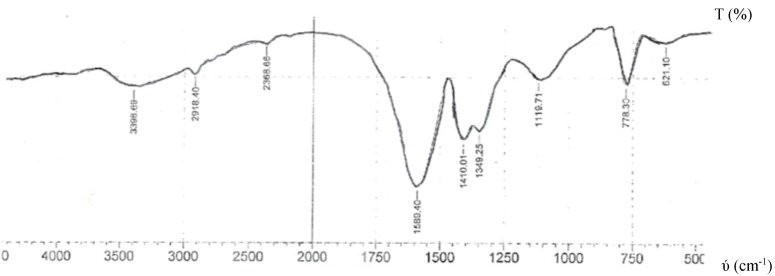
IR spectrum of [Nd(NBTCS)_2_(H_2_O)_2_]NO_3_.

The IR spectral data of NBTCS and its complexes are displayed in Table 3. The N–H stretching vibration for NBTCS was observed as a broad band at 3407 cm^−1^, this can be seen in Figure 2. However, in Nd(III) and Tl(III) complexes, this band disappeared. This suggests that the N–H was deprotonated prior to complexation. The IR spectrum of Nd(III) complex as shown in Figure 3 has a broad band at 3399 cm^−1^, assignable to water of hydration from the metal salt. This was further confirmed by the presence of a sharp peak at 778 cm^−1^ due to O–H bending vibrations. The peak observed around 3046 cm^−1^ in the Tl(III) complex was assigned to aromatic C–H stretching vibration. The peaks at 1545 and 1465 cm^−1^ in the ligand were assigned to C=N stretching vibration of benzothiazole ring. In the Nd(III) complex, these shifted to 1589 and 1410 cm^−1^ while in the Tl(III) complex a single peak at 1548 cm^−1^ was obtained. This suggest that the C=N of benzothiazole was involved in the coordination [21,29]. This was further supported by emergence of M–N bands between 392 and 420 cm^−1^. The medium peaks observed between 1154 and 1120 cm^−1^ in the compounds were assigned to SO_2_ stretching vibration. The observed decrease in wavenumber of the complexes up to 34 cm^−1^ suggests coordination through SO_2_ group. Similar observation has been made in the literature [44]. The Nd(III) complex showed new band at 1349 cm^−1^ due to ionic nitrate [45]. A medium band assigned to M–O stretch was observed in the Nd(III) complex at 621 cm^−1^. The medium bands at 953, 827, 750 and 845 cm^−1^ in the compounds were assigned to C–H bending vibration of substituted benzene ring [46]. The spectra of the compounds are in the Supplementary Materials.

### 2.3. ^1^H-NMR and ^13^C-NMR Spectra

In the ligand, the peaks obtained in the range 7.20–7.90 ppm are assigned to phenyl and benzothiazole protons. This appeared as a complex multiplet hence separate assignment could not be made. A single peak at 9.35 ppm is also assigned to benzothiazole protons. However, in the complexes, the peaks are clearly separated. In [Nd(NBTCS)_2_(H_2_O)_2_]NO_3_, the peak at 7.15 (2H, d) ppm and 7.35 ppm (2H, d) are assigned to phenyl protons while the peaks at the range of 7.60–8.20 ppm (4H, m) are assigned to benzothiazole protons. In [Tl(NBTCS)_2_]CH_3_COO, the peaks at 7.10 (2H, d) ppm and 7.25 (2H, d) ppm are assigned to the two chemically equivalent phenyl protons while the peaks at 7.28, 7.47, 7.52 and 7.79 ppm (4H, m) are assigned to benzothiazole protons. Similar observation has been made in literature [21,22,29,44,47]. The chemical shift due to the N–H proton is observed at 13.30 ppm for the ligand but disappeared in the metal complexes indicating its replacement by metal ions. In Tl(III) complex, peaks at 3.34 ppm (3H, s) are assigned to CH_3_ protons of acetate [22]. The spectra of all the compounds showed a multiplet centered at 2.50 ppm due to DMSO [46].

**Figure 4:**
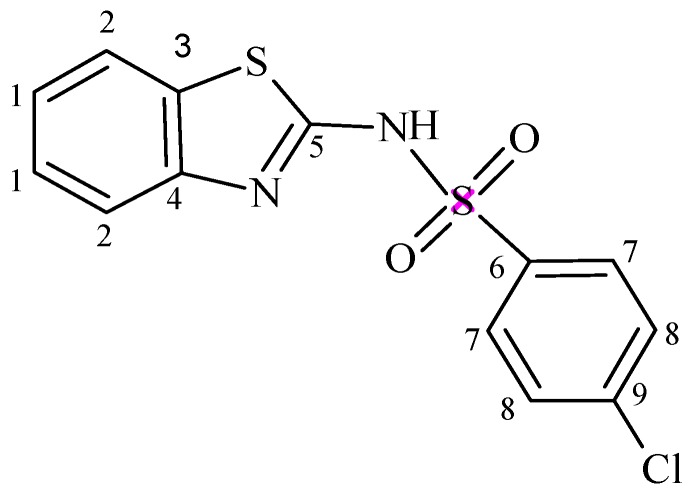
Structure of the ligand showing carbon numbering.

**Figure 5:**
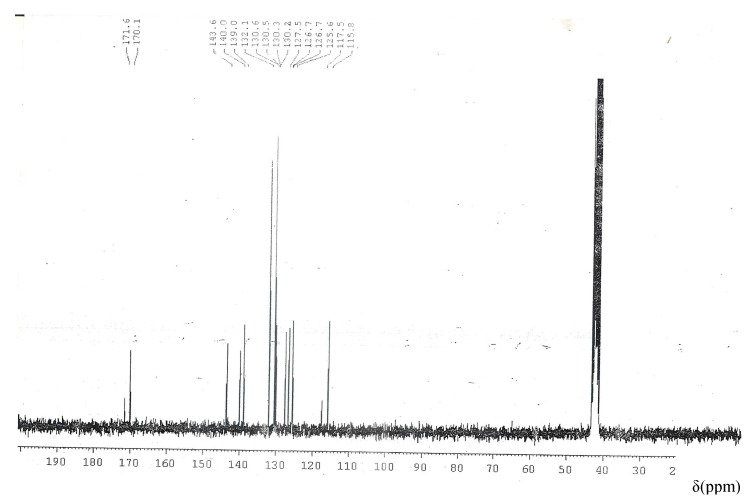
^13^C-NMR spectrum of NBTCS.

Figure 4 gives the structure of the ligand showing carbon numbering. ^13^C-NMR spectrum of the ligand as shown in Figure 5 has a peak at 115.80 ppm attributed to benzothiazole ring carbon (C1). Peaks assigned to benzothiazole ring carbon (C2) appear at 126.70 ppm. Peaks at 139.0 and 140.0 ppm are assigned to benzothiazole ring carbon (C3) and (C4) respectively. Peak at 170.10 ppm is attributed to benzothiazole ring carbon (C5). Peaks at 132.10 and 143.60 ppm are assigned to phenyl ring carbon (C6) and (C9) respectively. The increase in chemical shift of C6 and C9 are due to increase in the electronegativity of the atoms attached to them. C9 shows a higher value than C6 because chlorine which is attached to it is more electronegative than sulphur which is attached to C6. Peaks assignable to phenyl ring carbon (C7) and (C8) are observed at 130.30 and 130.60 ppm respectively. Similar observations have been made in literature [21,22,29,44]. The ^13^C-NMR of the complexes show none of the peaks except for the solvent peak at 40.0 ppm. The spectra of the compounds are in the Supplementary Materials.

**Figure 6:**
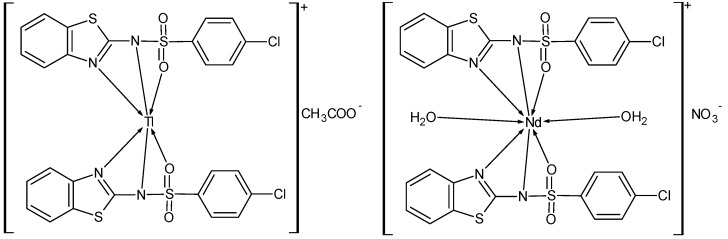
Proposed structure of the complexes.

Based on the spectral results, coordination numbers of eight and six were proposed for Nd(III) and Tl(III) complexes, respectively, as shown in Figure 6.

### 2.4. Antibacterial Activity

**Figure 7:**
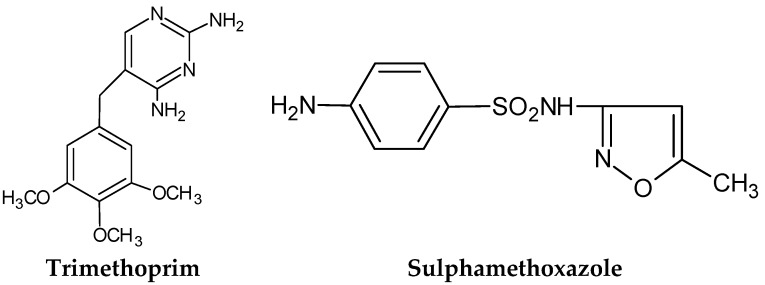
Structure of co-trimoxazole.

**Figure 8:**
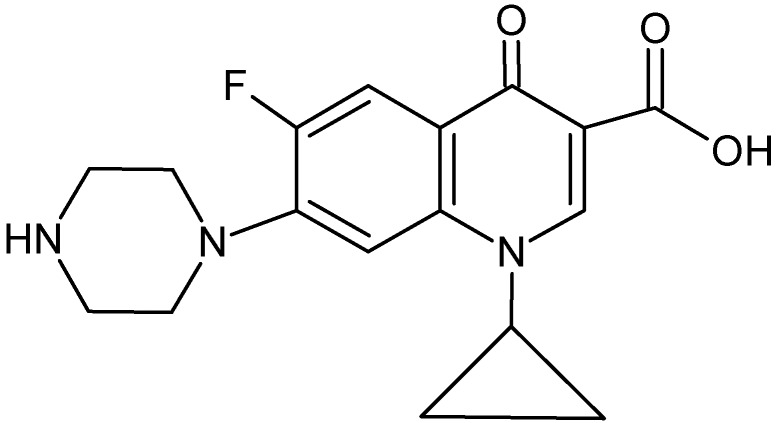
Structure of ciprofloxacin.

**Figure 9:**
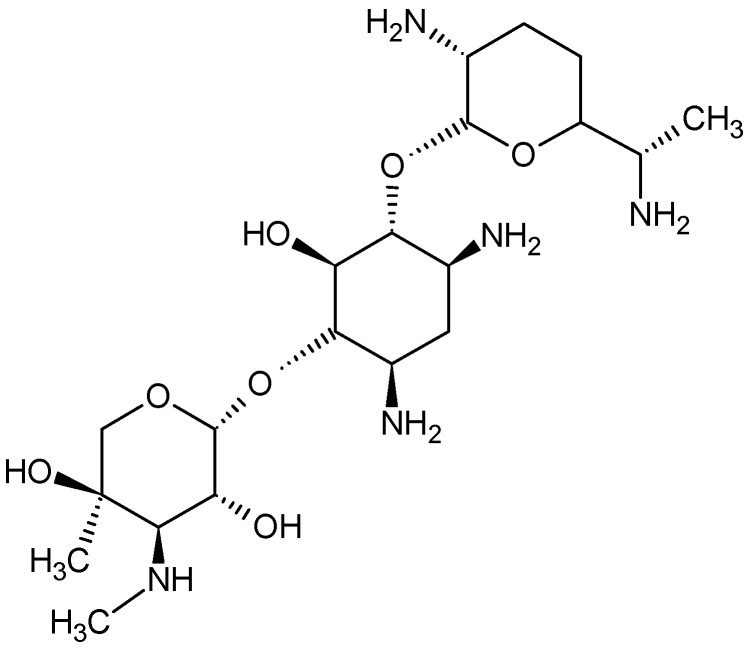
Structure of gentamicin.

The ligand and its complexes were screened in vitro for their antibacterial activity against multi-resistant bacterial strains isolated under clinical conditions namely: *Escherichia coli* strains (*E. coli 6* and *E. coli 13*), *Proteus* species*, Staphylococcus aureus* and *Pseudomonas aeruginosa*. The antibacterial activities of the compounds were determined using the agar well diffusion method [48]. The inhibitory zone diameter (IZD) and minimum inhibition concentration (MIC) in μg/mL of the compounds were determined. Co-trimoxazole, ciprofloxacin and gentamicin were used as positive control. The structure of these drugs are shown in Figure 7, Figure 8 and Figure 9 respectively. Gentamicin belongs to aminoglycosides and their mechanism of action is by inhibition of protein synthesis by preventing the synthesis of nucleic acids (DNA replication or RNA synthesis), thereby blocking transcription. Ciprofloxacin belongs to quinolones and inhibit bacteria growth by preventing DNA synthesis before mitosis [49]. Co-trimoxazole is a sulphonamide, known to prevent the growth of microorganisms by inhibiting folic acid production. Co-trimoxazole contains a fixed combination of sulphamethoxazole and trimethoprim.

The result of the IZD (Table 4) shows that the metal complexes exhibit higher antibacterial activity against the microorganisms relative to the ligand. It was also observed that the ligand did not inhibit the growth of *S. aureus* and *P. aeruginosa.* Likewise, the thallium complex had no effect on *P. aeruginosa.* The increase in antibacterial activity of the metal chelates might be due to the effect of the metal ion on the normal cell process. Chelation reduces the polarity of the metal ion because of partial sharing of its positive charge with donor groups and the π-electron delocalization over the whole chelate ring. Such chelation could enhance the lipophilic character of the central metal atom, which subsequently favours its permeation through the lipid layers of the cell membrane [50]. From the MIC values (Table 5), it is observed that the synthesized compounds are more active against *Escherichia coli* strains 6 and 13 relative to the standard drugs, since the lower the MIC value, the more effective the antibacterial activity of the drug. However, Ciprofloxacin has a higher antibacterial activity against *S. aureus* and *Proteus* species compared to the compounds. It can also be inferred from Table 5 that the synthesized compounds have better antibacterial activity against the microorganisms screened relative to Gentamicin and Co-trimoxazole.

## 3. Materials and Methods

### 3.1. Materials

All the reagents and solvents used were of analytical grade and were used as supplied unless otherwise stated. The melting point of the ligand and the complexes were determined using a Gallenkamp melting point apparatus (Weiss Technik, Loughborough, UK) and were uncorrected. Electronic spectra (in ethanol) were recorded on a UV-2500PC series spectrophotometer (SHIMADZU, Tokyo, Japan). The FTIR spectra were performed using FTIR-8400S Spectrophotometer (SHIMADZU), in the range 4500–200 cm^−1^ using KBr. The ^1^H- and ^13^C-NMR of the ligand were recorded on Bruker Spectrospin 250(Bruker, Billerica, MA, USA) using DMSO while the elemental analysis was done using Euro EA 3000 Dual CHNS Analyzer (Eurovector S.P.A, Zurich, Switzerland) both at the Department of Chemistry, University of Mauritius.

### 3.2. Synthesis of N-(Benzothiazol-2-yl)-4-chlorobenzenesulphonamide (NBTCS)

To a solution of 2-aminobenzothiazole (3.0 g; 20 mmol) in acetone (15 mL) was added 4-chlorobenzenesulphonylchloride (4.24 g; 20 mmol) with stirring. The mixture was refluxed for 45 min at 130 °C. A white precipitate was formed, which was recrystallized from absolute ethanol, dried and stored in a desiccator over anhydrous CaCl_2_. This is shown in Scheme 1. The yield was 40.83% and the melting point 220–222 °C.

### 3.3. Synthesis of [N-(Benzothiazol-2-yl)-4-chlorobenzenesulphonamide] Neodymium(III) Complex, [Nd(NBTCS)_2_(H_2_O)_2_]NO_3_

To a solution of *N*-(benzothiazol-2-yl)-4-chlorobenzenesulphonamide (1.62 g; 5.17 mmol) in acetone (10 mL) was added aqueous solution of neodymium(III) nitrate pentahydrate, Nd(NO_3_)_3_·5H_2_O (1.05 g; 1.37 mmol). This was refluxed for 1 h at 120 °C during which a yellow precipitate was formed. The resulting yellow precipitate formed was filtered, dried in a stream of air and stored in a desiccator over anhydrous CaCl_2_. The yield was 32.24% and the melting point is 245–247 °C.

### 3.4. Synthesis of [N-(Benzothiazol-2-yl)-4-chlorobenzenesulphonamide] Thallium(III) Complex, [Tl(NBTCS)_2_]CH_3_COO

To a solution of *N*-(benzothiazol-2-yl)-4-chlorobenzenesulphonamide (1.62 g; 5.17 mmol) in acetone (10 mL) was added aqueous solution of thallium(III) acetate, C_6_H_9_O_6_Tl (0.95 g; 1.14 mmol). This was refluxed for 1 h at 120 °C during which a brown precipitate was formed. The resulting brown precipitate formed was filtered, dried in a stream of air and stored in a desiccator over anhydrous CaCl_2_. The yield was 34.97% and the melting point is 235–237 °C.

### 3.5. Antibacterial Activity

The inhibitory activity of the ligand and its metal complexes were carried out on the following microorganisms: *Escherichia coli* strains (*E. coli 6* and *E. coli 13*), *Proteus* species*, Staphylococcus aureus* and *Pseudomonas aeruginosa.* The bacteria strains used were isolated from clinical conditions. The method used was the agar well-diffusion Technique [46]. Mueller-Hinton agar plates were inoculated with 0.1 mL of 3 h broth culture of the test microorganisms. Using a cork borer, wells (7 mm in diameter and 2.5 mm deep) were bored into the inoculated agar. Fresh solutions (1000 μg/mL) of all the synthesized compounds were prepared in DMSO and 50 μL of each compound was delivered into the wells. Wells containing 1000 μg/mL of ciprofloxacin, gentamicin and co-trimoxazole in DMSO were used as positive controls. The plates were incubated at 37 °C for 24 h and assessment of antibacterial activity was based on the measurement of the diameter of inhibition zone (IZD) around the wells.

The minimum inhibitory concentrations (MIC) of the test compounds were determined using the same method. Two-fold serial dilutions of test compound were made in DMSO. The final concentration of the compounds ranged from 250 to 0.625 μg/mL. Inoculated plates were incubated at 37 °C for 24 h and observed for presence of visible growth. The minimum inhibition concentration was determined as the value of the lowest concentration that completely suppressed the growth of the microorganisms.

## 4. Conclusions

NBTCS and its neodymium(III) and thallium(III) complexes were synthesized. Based on the UV, IR and NMR spectral studies and elemental analysis, a coordination number of eight was proposed for neodymium(III) complex and six for thallium(III) complex. The antibacterial activity revealed that the synthesized compounds have better antibacterial activity against the microorganisms screened relative to gentamicin and co-trimoxazole. The low MIC values observed for NBTCS and its complexes indicate the potency of these compounds as antibacterial agents. This serves as a marker in drug design, hence these compounds should be screened for in vivo antibacterial activity and the lethal dose (LD_50_) determined.

## Figures and Tables

**Table 1:** Physical properties and elemental analysis of *N*-(Benzothiazol-2-yl)-4-chlorobenzenesulphonamide (NBTCS) and its complexes.

Compound	Molar Weight (g/mol)	Cal. (Exp., %)				Molar Cond. (Ω^−1^·cm^2^·mol^−1^)	Colour	Yield (%)
		C	H	N	S			
NBTCS	324.5	48.07	2.77	8.63	19.72	15.6	White	40.83
		(48.11)	(2.94)	(8.75)	(19.89)			
[Nd(NBTCS)_2_(H_2_O)_2_]NO_3_	891	35.02	2.47	7.86	14.37	72	Yellow	32.24
		(34.99)	(2.36)	(7.60)	(14.20)			
[Tl(NBTCS)_2_]CH_3_COO	912	36.84	2.30	6.14	14.04	88	Brown	34.97
		(36.40)	(2.80)	(6.07)	(13.96)			

**Table 2:** UV-Vis spectral data λ_max_ (nm) of NBTCS and complexes.

Compound	λ_max_ (nm)	ῦ (cm^-1^)	ε (L·mol^-1^·cm^-1^)	Assignment
NBTCS	272, 282	29,239, 30,314	6925, 6860	π–π*
	305	32,787	7363	n–π*
[Nd(NBTCS)_2_(H_2_O)_2_]NO_3_	230, 237, 250	24,725, 25,477, 26,875	8237,8333,8333	π–π*
[Tl(NBTCS)_2_]CH_3_COO	241, 248, 261	25,907, 26,659, 28,057	8333,8333,8333	π–π*

**Table 3:** Infrared spectral data for NBTCS and its complexes.

Compound	ν_N–H_ (cm^−1^)	ν_O–H_ (cm^−1^)	ν_C–H_ (cm^−1^)	ν_C=N_ (cm^−1^)	ν_SO2_ (cm^−1^)	ν_NO3_ (ionic) (cm^−1^)	δ_C–H_ (cm^−1^)	δ_O–H_ (cm^−1^)	ν_M–O_ (cm^−1^)	ν_M–N_ (cm^−1^)
NBTCS	3407	-	-	1545 1465	1154		953, 827, 750	-	-	-
[Nd(NBTCS)_2_(H_2_O)_2_]NO_3_	-	3399	-	1589 1410	1120	1349	-	778	621	392
[Tl(NBTCS)_2_]CH_3_COO	-	-	3142	1548	1128	-	845		-	420

ν = stretching vibrations, δ = bending vibrations.

**Table 4:** Antibacterial activity of NBTCS, [Nd(NBTCS)_2_(H_2_O)_2_]NO_3_ and [Tl(NBTCS)_2_]CH_3_COO.

IZD Values (mm)					
Compounds	*E. coli 6*	*E. coli 13*	*S. aureus*	*P. aeruginosa*	*Proteus* species
NBTCS	13	10	0	0	12
[Nd(NBTCS)_2_(H_2_O)_2_]NO_3_	15	14	17	12	16
[Tl(NBTCS)_2_]CH_3_COO	12	12	15	0	12

IZD: inhibitory zone diameter.

**Table 5:** MIC values of the compounds and standard drugs.

MIC Values (μg/mL)					
Compounds	*E. coli 6*	*E. coli 13*	*S. aureus*	*P. aeruginosa*	*Proteus* species
NBTCS	3.16	4.57	0.00	0.00	3.39
[Nd(NBTCS)_2_(H_2_O)_2_]NO_3_	1.78	2.19	1.45	3.31	1.26
[Tl(NBTCS)_2_]CH_3_COO	3.47	3.47	1.26	0.00	3.47
Ciprofloxacin	20.00	20.00	0.63	10.00	0.16
Gentamicin	100.00	100.00	2.50	20.00	5.00
Co-trimoxazole	100.00	100.00	50.00	170.00	30.00

MIC: minimum inhibition concentration; 0.00 = no antibacterial activity.

## Supplementary Materials

The following are available online at: http://www.mdpi.com/1420-3049/22/2/153/s1, FTIR Spectra, electronic spectra, ^1^H-NMR and ^13^C-NMR of the ligand and complexes.
