# Supplementary material for: Synthesis, Characterization and Antibacterial Studies of N-(Benzothiazol-2-yl)-4-chlorobenzenesulphonamide and Its Neodymium(III) and Thallium(III) Complexes
Source: Molecules. 2017 Feb 22;22(2):153. doi: 10.3390/molecules22020153 (PMC6155816; doi:10.3390/molecules22020153)
Supplement: Supplementary file 1 [file molecules-22-00153-s001.pdf]

Supplementary Materials: Synthesis, Characterization and Antibacterial Studies of *N*-(Benzothiazol-2-yl)-4-chlorobenzenesulphonamide and Its Neodymium(III) and Thallium(III) Complexes

Lawrence Nnamdi Obasi, Uchechukwu Susan Oruma, Ibrahim Abdulrazak Al-Swaidan, Ponnadurai Ramasami, Chigozie Julius Ezeorah and Alfred Ezinna Ochonogor

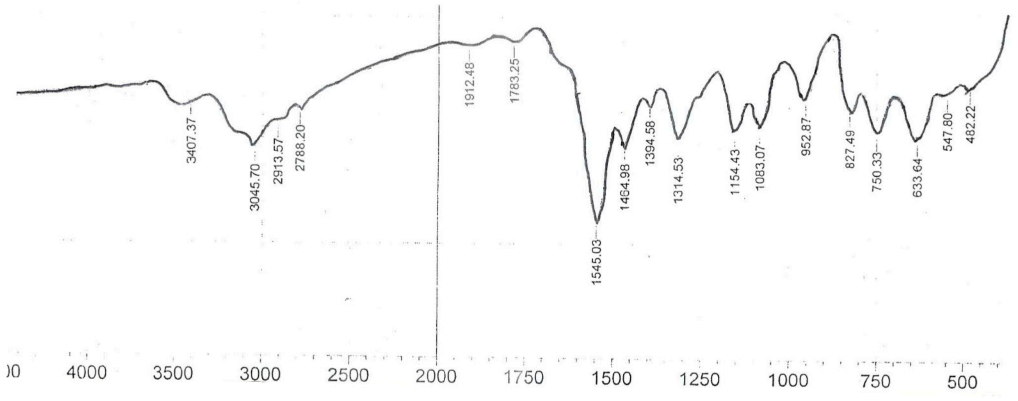

Figure S1 FTIR Spectrum of NBTCS.

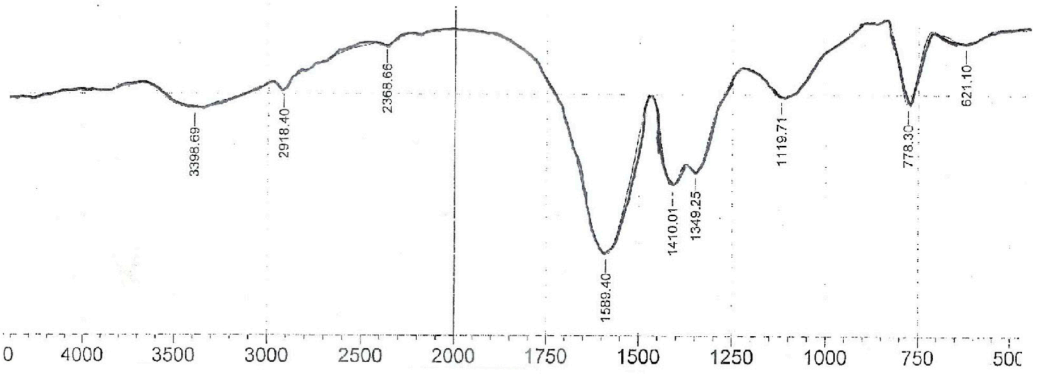

Figure S2. FTIR Spectrum of $[Nd(NBTCS)_2(H_2O)_2]NO_3$.

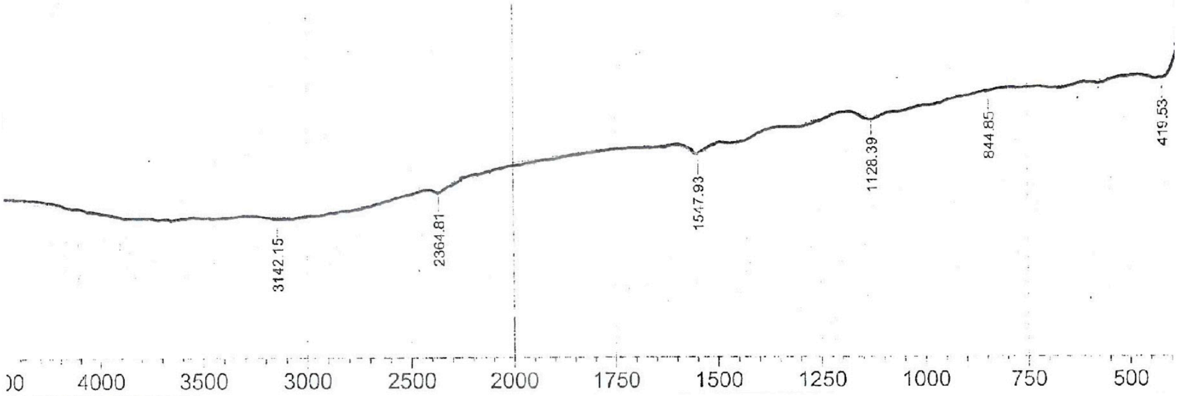

Figure S3. FTIR Spectrum of $[Tl(NBTCS)_2]CH_3COO$.

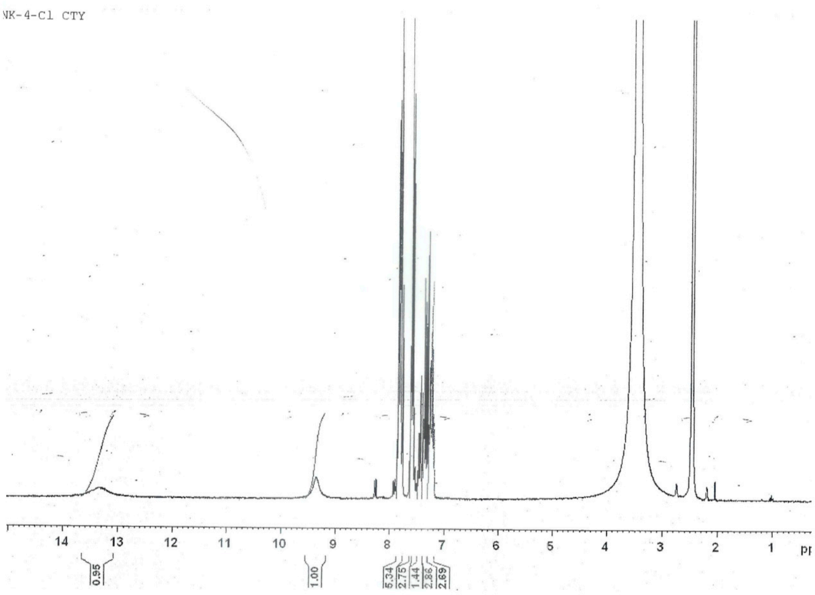

Figure S4. ^{1}H-NMR Spectrum of NBTCS.

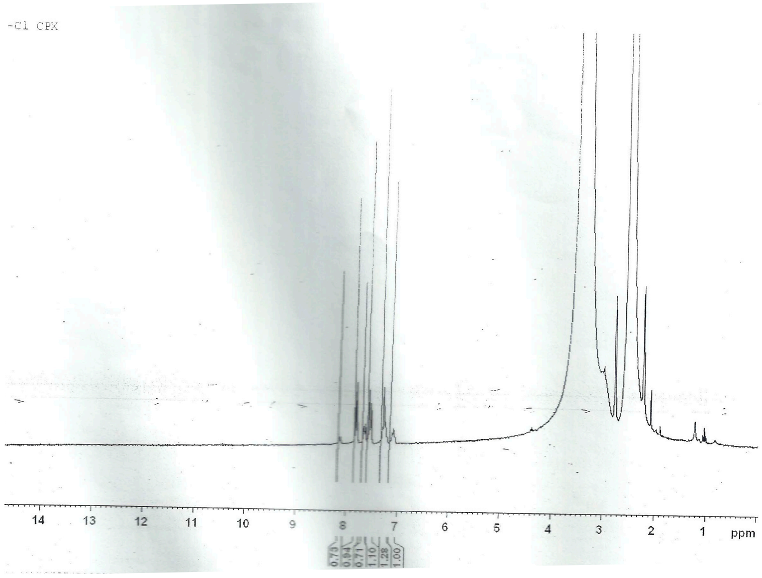

Figure S5. ^{1}H-NMR Spectrum of $[Nd(NBTCS)_2(H_2O)_2]NO_3$.

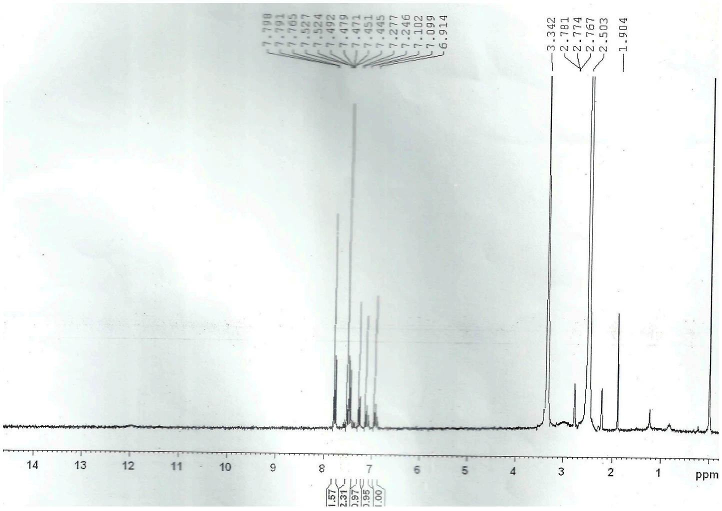

Figure S6. ^{1}H-NMR Spectrum of $[Tl(NBTCS)_2]CH_3COO$.

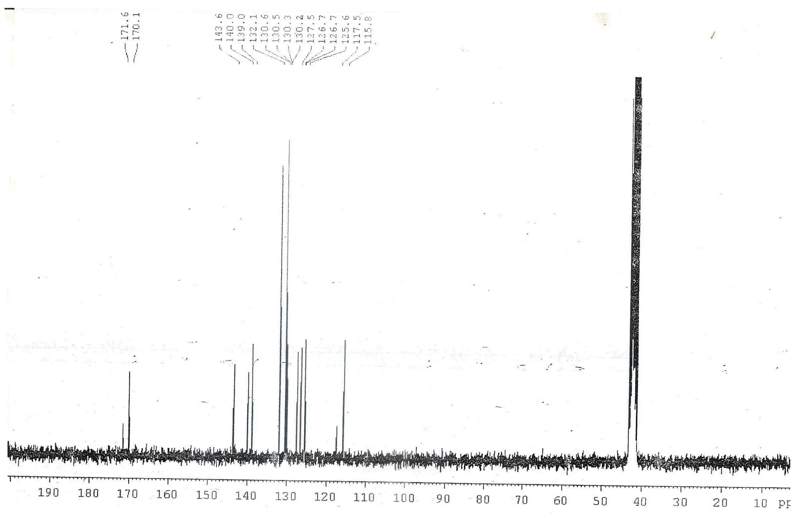

Figure S7. ^{13}C-NMR Spectrum of NBTCS.

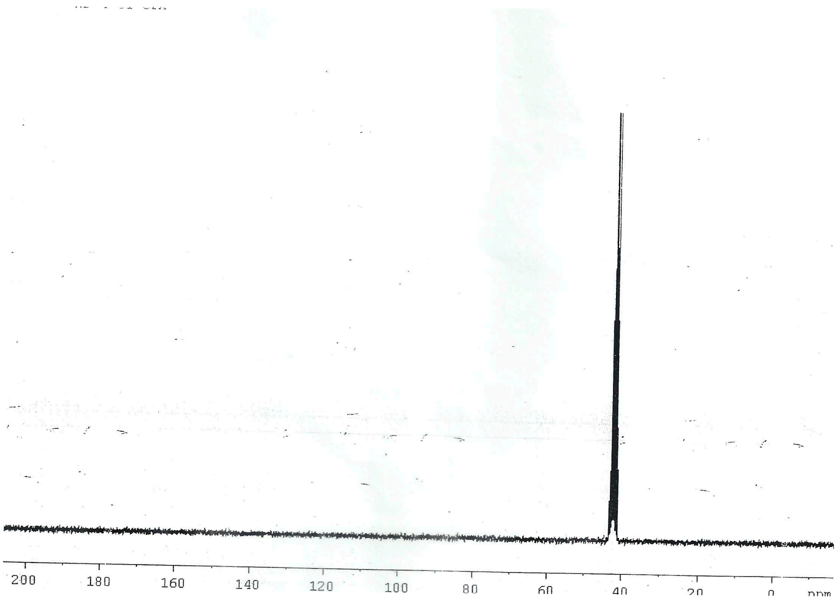

Figure S8. ^{13}C-NMR Spectrum of $[Nd(NBTCS)_2(H_2O)_2]NO_3$.

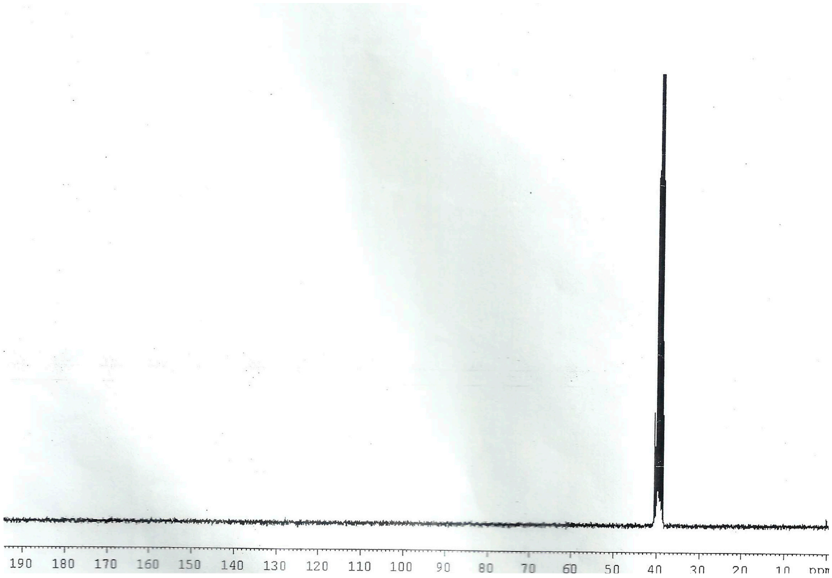

Figure S9. ^{13}C-NMR Spectrum of $[Tl(NBTCS)_2]CH_3COO$.

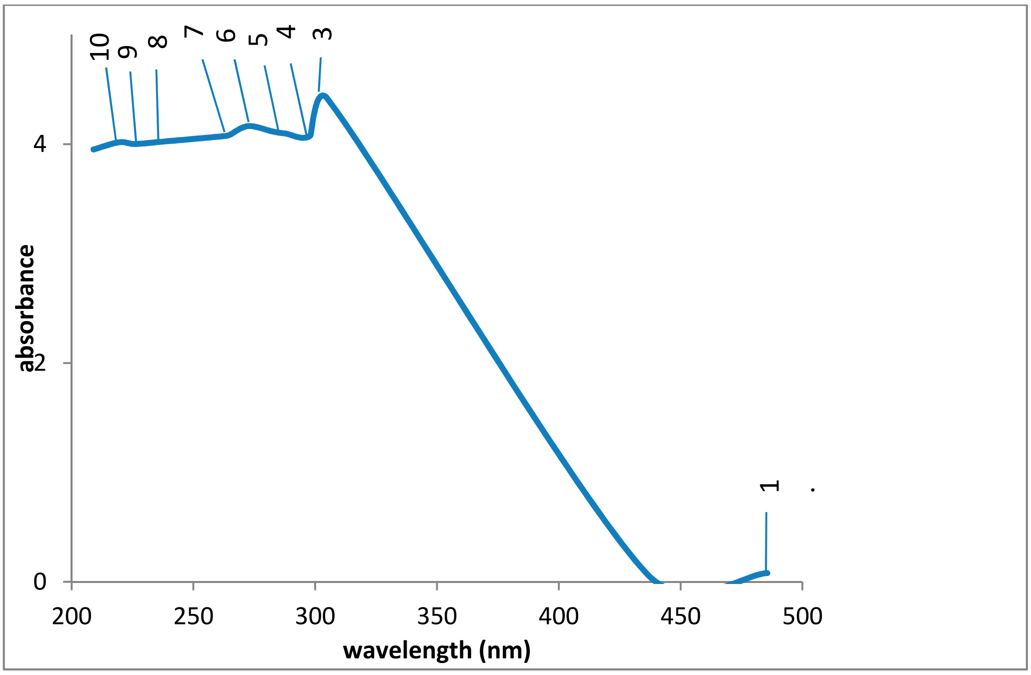

λ (nm)	Absorbance	Plot
485.50	0.080	1
436.00	0.078	2
305.00	4.418	3
298.00	4.078	4
287.50	4.098	5
282.50	4.116	6
272.50	4.155	7
265.00	4.086	8
253.00	4.074	9
235.50	4.019	10
226.00	4.001	11
219.50	4.017	12
209.00	3.951	13

Figure S10. UV-Visible Spectrum of NBTCS.

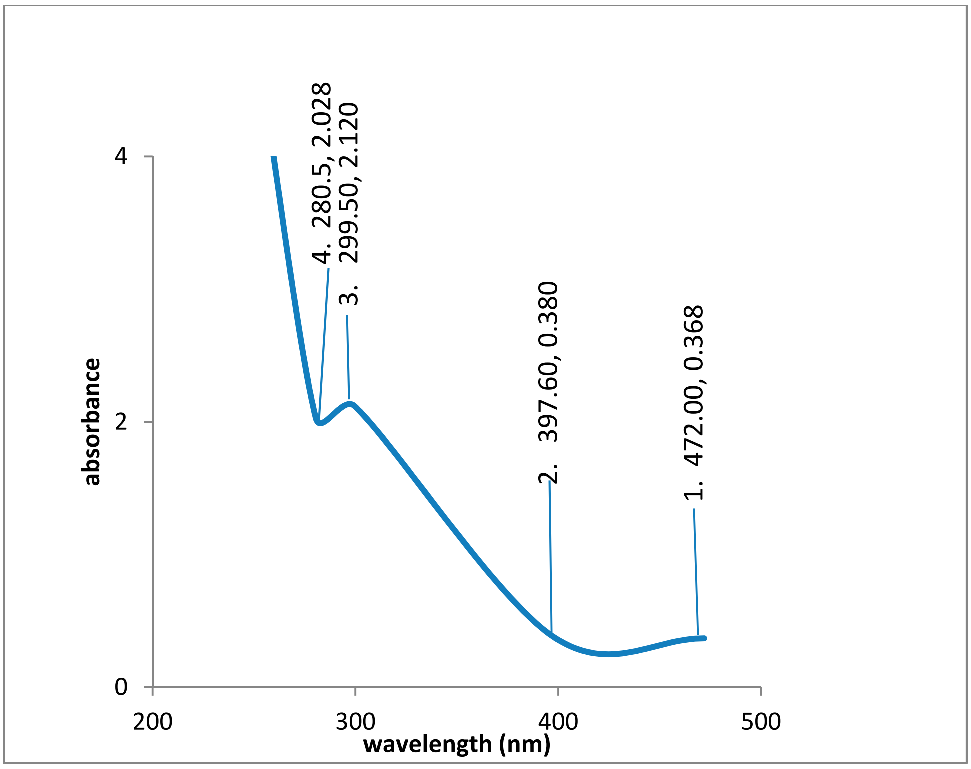

λ (nm)	Absorbance	Plot
472.00	0.368	1
397.50	0.380	2
299.50	2.120	3
280.50	2.028	4
250.00	5.000	5
237.00	5.000	6
230.50	4.942	7
224.50	4.923	8
218.50	4.8110	9
211.50	4.808	10

Figure S11. UV-Visible Spectrum of $[Nd(NBTCS)_2(H_2O)_2]NO_3$.

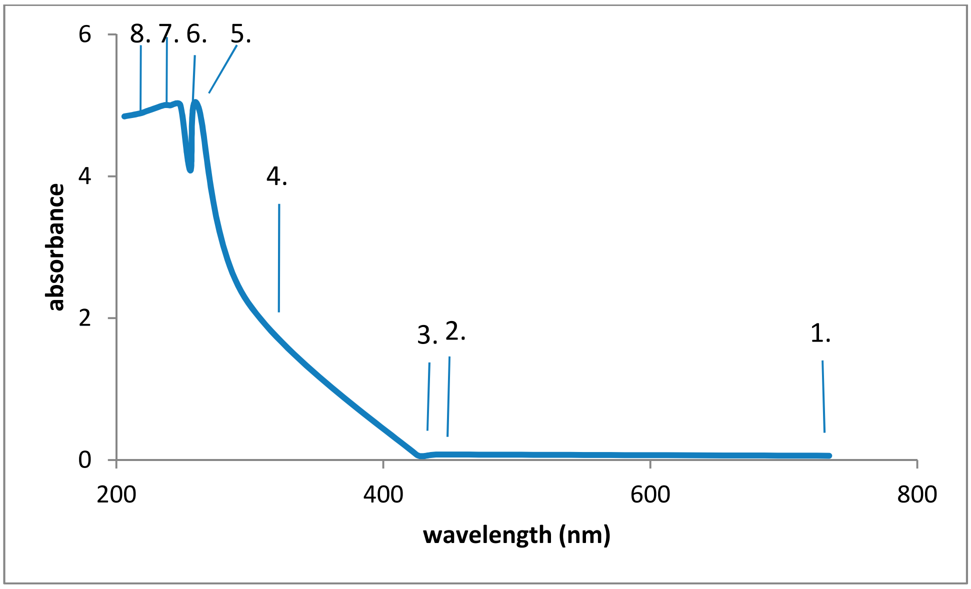

λ (nm)	Absorbance	Plot
734.00	0.060	1
441.00	0.078	2
424.50	0.081	3
296.00	2.297	4
261.00	5.000	5
255.50	4.081	6
248.00	5.000	7
240.50	5.000	8
235.50	5.000	9
223.50	4.923	10
217.00	4.883	11
206.00	4.844	12

Figure S12. UV-Visible Spectrum of [Tl(NBTCS)$_2$]CH_3COO.
